# Editorial: Multimorbidity in primary care

**DOI:** 10.3389/fmed.2024.1401711

**Published:** 2024-03-25

**Authors:** Stefan Essig, Sanghamitra Pati

**Affiliations:** ^1^Center for Primary and Community Care, University of Lucerne, Lucerne, Switzerland; ^2^Interface Policy Studies Research Consulting, Lucerne, Switzerland; ^3^ICMR-Regional Medical Research Centre, Bhubaneswar, India

**Keywords:** primary care (MeSH), multimorbidity, coordination of care, polypharmacy (MeSH), access and quality, patient centered

## Background

Multimorbidity, the coexistence of two or more chronic health conditions accompanied by reduced quality of life and increased healthcare utilization, and expenditure is a significant public health challenge. The prevalence of multimorbidity is on the rise, primarily due to an aging population, leading to diminished quality of life and life expectancy, along with an increased reliance on emergency or unplanned healthcare services. Managing multimorbidity requires continuous and coordinated care, thus posing substantial challenge for health systems and patients alike. The World Health Organization underscores the pivotal role of primary care in addressing multimorbidity (1). Primary care often being the first and frequent point of contact for patients with multimorbidity, has a critical role in providing quality of care for these individuals. In order to address the holistic needs of these population, primary care strengthening should be one of the key health system priorities. Toward this, understanding the role of primary care in effectively managing multimorbidity is essential for optimizing patient outcomes, enhancing healthcare delivery efficiency, and promoting patient-centered care.

## Research

This editorial summarizes the contributing articles to the Research Topic “*Multimorbidity in primary care*.” We received a total of 38 manuscripts and were able to approve twelve of them for publication. Out of the twelve articles, eleven were original research and one was a perspective.

Overall, the studies collectively emphasize the intricate relationship between multimorbidity, healthcare delivery, patient experiences, and health outcomes in diverse populations and settings, underscoring the importance of understanding these dynamics to develop effective strategies for addressing the challenges posed by multimorbidity and enhancing patient care and outcomes.

Zhou et al. delved into the growing public health concern of hypertension in China, particularly examining self-care compliance, trust, and satisfaction among hypertensive patients. The study revealed a positive association between trust, satisfaction, and compliance, with insights that can guide interventions to enhance hypertension management. A similar study by Yun et al. examined the effects of regional healthcare disparities on complications in hypertensive patients in South Korea which highlighted the importance of identifying disparities to improve healthcare access and outcomes.

Su et al. addressed the public health issue of multimorbidity coexistence in older adults in China, examining its effects on all-cause mortality. The study, spanning a 10-year period, found that multimorbidity is associated with an increased risk of death in older individuals, with a more significant effect in those aged 80–94 years. Age heterogeneity is observed, emphasizing the need for tailored interventions to address multimorbidity in different demographic groups. Palo et al. assessed the prevalence and patterns of multimorbidity among chronic kidney disease patients in India, highlighting the need for regular screening and management of associated chronic conditions. Eyowas et al. found in Northwest Ethiopia that multimorbidity patients attending chronic outpatient care had higher rates of developing new conditions, hospitalization, and mortality; suggesting the need for future studies to understand the multimorbidity trajectories. Another study group in Ethiopia, Bambo et al., determined the prevalence and factors associated with postpartum anemia.

Craig et al. investigated the link between multimorbidity and health-related quality of life (HRQoL) in Jamaica. Using latent class analysis, the study identified four multimorbidity classes and assessed their impact on physical and mental dimensions of HRQoL, highlighting the differential effects of specific disease combinations and the mediating role of health service use. Furthermore, Lee et al. observed in Australia that functional limitation acts as a mediator between multimorbidity and HRQoL, underscoring the importance of improving functional status in patient care.

Onaisi et al. focused on statin prescription for cardiovascular primary prevention in primary care settings in the French region of Rhône-Alpes, examining the association between multimorbidity and appropriate statin prescription. The study found that multimorbidity alone does not determine appropriate statin prescription; rather, the presence of diabetes influenced prescription decisions, highlighting the importance of differentiating between diabetic and non-diabetic multimorbidity for improved prevention. Lüthi-Corridori et al. conducted a study to identify factors predicting length of hospital stay, mortality, and re-hospitalization within 6 months for patients admitted with pulmonary embolism in a Swiss hospital. They found that diabetes, among other factors, was associated with longer hospital stays. The study suggests that understanding these factors can aid clinicians.

van Pinxteren et al. explored the social dimensions of multimorbidity management among vulnerable populations in South Africa, indicating that the treatment burden and capacity for patients is a crucial step to redesign health systems. The perspective article of Sagan et al. shed light on the challenges faced by Central and Eastern European countries in catering to complex patients with chronic conditions and multimorbidity. Despite relatively younger populations, these countries experience high prevalence of chronic conditions, and the study identified key initiatives to improve care coordination, emphasizing the need for progress and learning from both successful and failed attempts.

## Summary

To summarize, multimorbidity is a complex issue that affects patient-reported outcome measures, healthcare delivery, and patient experiences. Strengthening primary care would enable multimorbidity management through a holistic approach and may be the epicenter to provide continued and coordinated person-centered care. Primary healthcare providers often consider a holistic approach, taking into account individual diseases and their interactions, allowing for effective concurrent management of multiple morbidities, and resultant polypharmacy. Primary care being easily accessible provides a scope for better monitoring and prevention of chronic conditions and allows for timely interventions. Primary care providers prioritize patient involvement in decision-making, resulting in smooth transitions between specialties and settings. They also emphasize preventive care and health promotion, addressing modifiable risk factors, encouraging healthy behaviors, and providing routine screenings, all of which lead to better health outcomes and a higher quality of life for people with multimorbidity. We believe that these findings will assist researchers in better understanding multimorbidity in primary care.

## Author contributions

SE: Writing – original draft, Writing – review & editing. SP: Writing – original draft, Writing – review & editing.
